# Band Gap Tuning in 2D Layered Materials by Angular Rotation

**DOI:** 10.3390/ma10020147

**Published:** 2017-02-08

**Authors:** Javier Polanco-Gonzalez, Jesús Alfredo Carranco-Rodríguez, José L. Enríquez-Carrejo, Pierre G. Mani-Gonzalez, José Manuel Domínguez-Esquivel, Manuel Ramos

**Affiliations:** 1 Departamento de Física y Matemáticas, Instituto de Ingeniería y Tecnología, Universidad Autónoma de Cd. Juárez, Avenida del Charro #450 N. Cd. Juárez, Chihuahua C.P. 32310, Mexico; javier.polanco@uacj.mx (J.P.-G.); jose.enriquez@uacj.mx (J.L.E.-C.); pierre.mani@uacj.mx (P.G.M.-G.); 2 Materials Science and Engineering, McMaster University, 1280 Main St. West, Hamilton, ON L8S4L7, Canada; carrancj@mcmaster.ca; 3 Instituto Mexicano del Petróleo, Eje Central Lázaro Cárdenas No 152, México D.F. 07730, Mexico; jmdoming@imp.mx

**Keywords:** Moiré patterns, MoS_2_, graphene, WS_2_, WSe_2_, HRTEM

## Abstract

We present a series of computer-assisted high-resolution transmission electron (HRTEM) simulations to determine Moiré patters by induced twisting effects between slabs at rotational angles of 3°, 5°, 8°, and 16°, for molybdenum disulfide, graphene, tungsten disulfide, and tungsten selenide layered materials. In order to investigate the electronic structure, a series of numerical simulations using density functional methods (DFT) methods was completed using Cambridge serial total energy package (CASTEP) with a generalized gradient approximation to determine both the band structure and density of states on honeycomb-like new superlattices. Our results indicated metallic transitions when the rotation approached 8° with respect to each other laminates for most of the two-dimensional systems that were analyzed.

## 1. Introduction

Layered materials have attracted much attention lately, because of their exceptional catalytic, photovoltaic and semiconducting properties [1,2]. An extensive characterization for 2D materials such as molybdenum disulfide (MoS_2_), tungsten disulfide (WS_2_), tungsten diselenide (WSe_2_) and graphene has occurred in the past decades, using field emission gun microscopic techniques, such as scanning electron [3], transmission electron [4] and tunneling microscopy [5] with high accuracy. Observations reveal in detail their layered sandwich structure consisting of S-Mo(W)-S and Se-W-Se layers held together at a distance of 0.62 nm by weak Van der Waals forces [6]. The formation of Moiré patterns in those particular layered materials was observed by Kobayashi using scanning tunneling microscopy [5], and by Yacaman implementing the transmission electron microscopy technique [7], both concluding that the formation of Moiré patterns is attributed to strain effects between layered slabs with respect to the (001) crystallographic direction, creating honeycomb-like structures. Additionally, Jasinski observed similar Moiré patterns for single layers of graphene attributed to a 5° of rotation between slabs [8]. Recently, we were able to determine the electronic band structure of strained 2R-MoS_2_ (SG-R3m) when it is rotated about 10° (with respect to the (001)-basal plane). Our density functional theory (DFT) calculations determined a rapid semiconducting-to-metallic state transition, as indicated by two irreducible points near K → Γ and A → L when sampling over the Brillouin zone with Fermi wave vector values of ***k_F_*** at ~0.47 Å^−1^ and ***k_F_*** at ~0.37 Å^−1^ near 10° to 12° of rotation; for more details, we invite the reader to review in detail our manuscript as indicated in Reference [9]. However, the point we would like to introduce here is the formation of a new lattice parameter, which is a new O-lattice formed by the rotation of crystallographic structures as described by Bollman in late 1970s [10] and presented by Remskar [11] and Gomez and Romeu for the reciprocal lattice rotation between two crystals [12]. Creating this natural strain between slabs is relatively easy due to the weak Van der Waals bonding between the slabs, and this is the reason why it was possible to create a single layer of graphene as presented by Geim and Novoselov et al. [13]. Zhang et al. were able to achieve tuning of single layers of the graphene band gap when fabricating a field-effect transistor [14]; thus, we present a series of new O-lattices for honeycomb-like structures as formed by rotating 2D layered structures with respect to the (001) basal plane, along with a series of computer-assisted electronic structure calculations using density functional methods to identify if there is any transition (tuning) from semiconducting to metallic and vice versa on those specific nanostructure materials.

## 2. Results and Discussion

**Figure 1:**
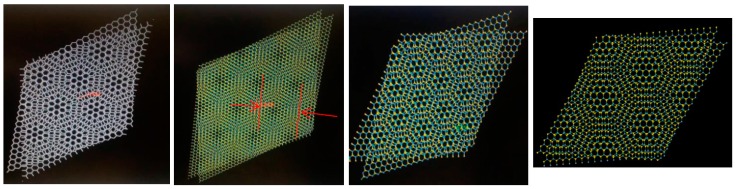
Moiré pattern as formed by angular rotation of 2D layered materials molecular models. From left to right: graphene, tungsten diselenide (WSe_2_), tungsten disulfide (WS_2_) and molybdenum disulfide (MoS_2_) at 19° of rotation.

**Figure 2:**
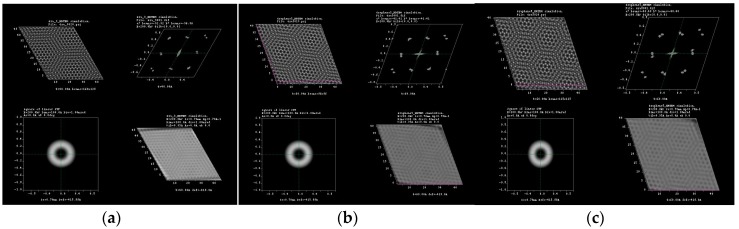
HRTEM simulation for (**a**) 3°, (**b**) 8° and (**c**) 16° of rotation in graphene molecular structure.

**Figure 3:**
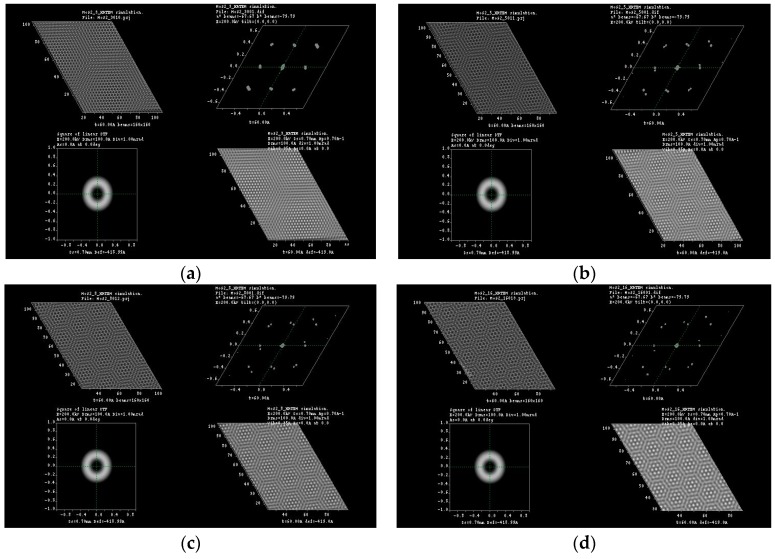
HRTEM simulation for (**a**) 3°; (**b**) 5°; (**c**) 8° and (**d**) 16° of rotation in molybdenum disulfide molecular structure.

**Figure 4:**
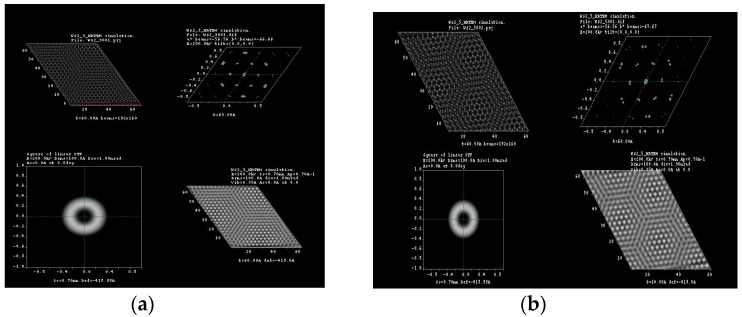

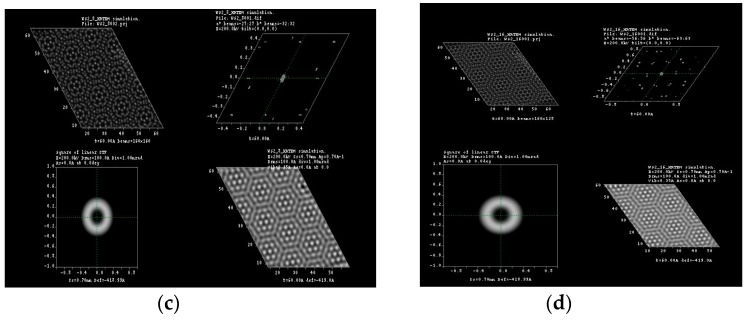
HRTEM simulation for (**a**) 3°; (**b**) 5°; (**c**) 8° and (**d**) 16° of rotation in tungsten disulfide molecular structure.

**Figure 5:**
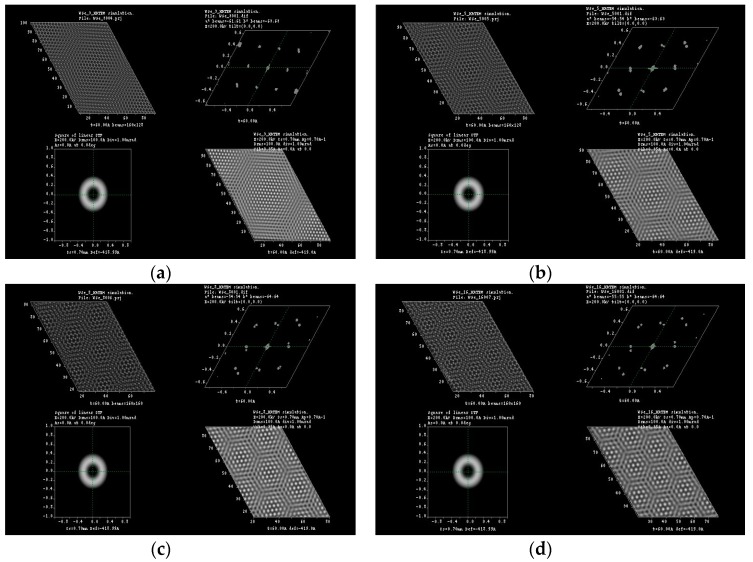
HRTEM simulation for (**a**) 3°; (**b**) 5°; (**c**) 8° and (**d**) 16° of rotation in tungsten diselenide molecular structure.

Typical Moiré patterns are observed when two 2D lattices are rotated at a certain angle, and this observation is made directly at their basal plane, usually called (001), over the *c*-axis direction. We reported these observations previously in experimental HRTEM and simulated them for 2R-MoS_2_ slabs when rotated about 12° in the *c*-direction [3,9]. Furthermore, we were able to produce the formation of Moiré patterns on other materials (i.e., tungsten disulfide (WS_2_), graphene and tungsten diselenide (WSe_2_)) by achieving this lattice rotation on the molecular models, which created honeycomb-like features, observing as well the formation of new O-lattices as presented in Figure 1 (corresponding to the distance between ending points at the edge of the honeycomb-like structure). Later, those molecular models were subjected to HRTEM simulations, varying *a*, *b* coefficients in the projected potential as described by Equation (1) in Section 3.1. The as-simulated sequence of images is presented in Figure 2, Figure 3, Figure 4 and Figure 5; it is possible to determine the formation of a large O-lattice with a non-defined honeycomb structure for angles of rotation corresponding to 3° and a strong honeycomb structure at 16°. Moreover, the resulting O-lattice was measured digitally using the BIOVIA^®^ Materials Studio crystal builder tool. Our findings are presented (for easy reading) in Table 1 and graphically by the inset of red arrows in Figure 1.

It is important to mention that Moire patterns are not typically seen by HRTEM techniques for bulk 2D-layered materials; for example, when surveying 2R-MoS_2_ slabs, fringes are commonly observed and it is possible to determine the Van der Waals force gap of 6.2 Å between slabs [8] and also to see clearly that synthesis conditions can cause the “turbostraticity” bending of the layered structure. Thus, one must have cautious procedures when fabricating nanoscale-organized devices [9]. Therefore, analysis of the electronic structure corresponding to rotated 2D layered structures needs to be performed. The electronic structures of these O-lattices, as calculated by DFT methods, reflect a rapid transition for the majority of these layered structures; our findings are presented in Figure 3.

**Figure 6:**
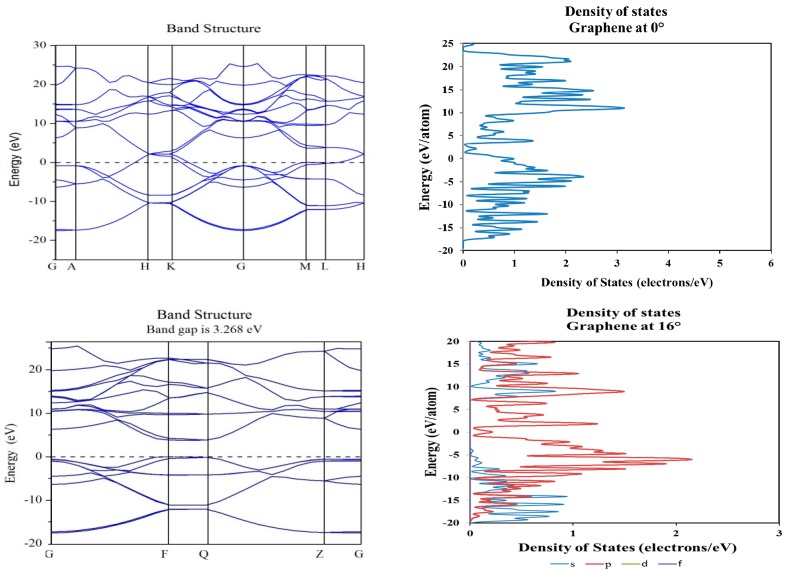
Band structure and density of states plots corresponding to 0° (**top**) and 16° (**bottom**) of rotation in graphene molecular structure; a tuning on the electronic band gap is observed near the Fermi level (0 eV).

**Figure 7:**
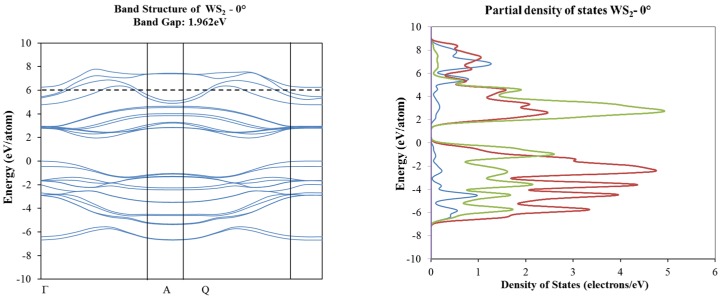

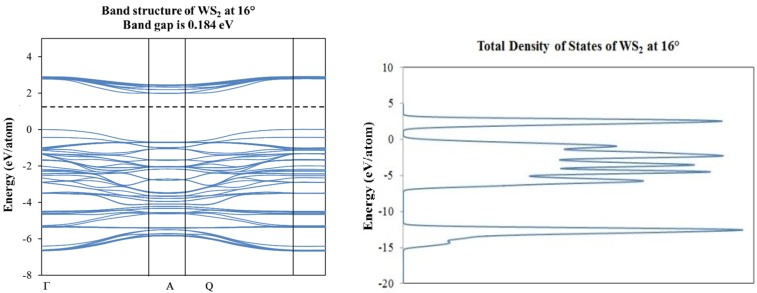
Band structure and partial total density of states plots corresponding to 0° (**top**) and 16° (**bottom**) of rotation in WS_2_ molecular structure.

**Figure 8:**
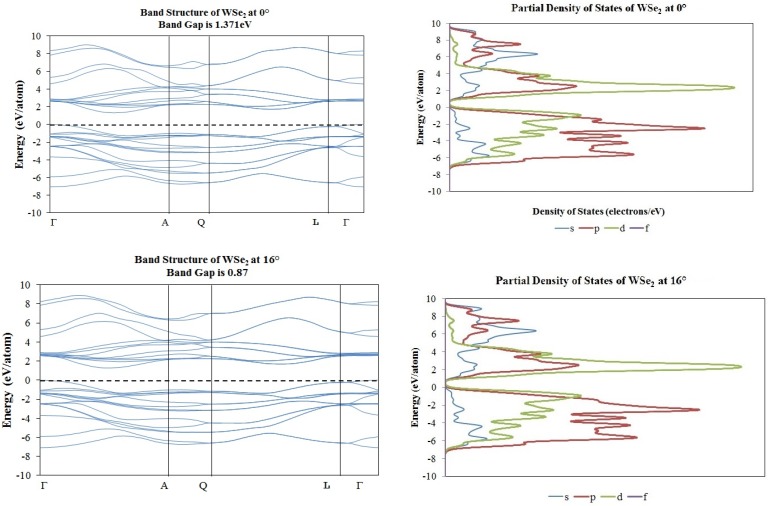
Band structure and partial density of states plots corresponding to 0° (**top**) and 16° (**bottom**) of rotation in WSe_2_ molecular structure.

In order to determine the electronic structure of those new O-lattices as created by the angular rotation of the molecular structures, we proceeded with band structures using CASTEP, as described in Section 3.2 and elsewhere [9]. Our results indicated some transitions from metallic into a wide-band-gap semiconductor for the graphene layers, as presented by the band structure and density of states corresponding to 0° and 16° shown in Figure 6. The opposite occurs for the case of the WS_2_ and WSe_2_ and MoS_2_ (presented in Reference [9]) molecular structures, as presented in Figure 7 and Figure 8.

**Figure 9:**
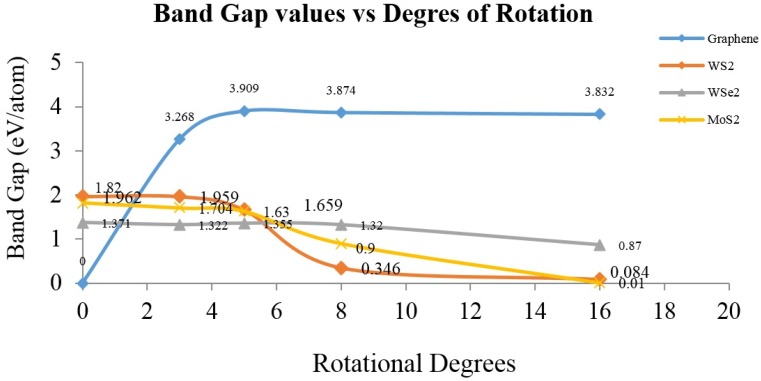
Band gap values of layered molecular models corresponding to graphene, WS_2_, WSe_2_ and MoS_2_, as obtained from DFT numerical simulations using CASTEP algorithm.

Because of the large data calculations, all band gap values were added and plotted with the degree of rotation, as presented in Figure 9 in band gap vs. degrees of rotation. It is possible to achieve appropriate conclusions by using this fast and low-cost approach helps understand Bollman O-lattice formation due to the “twisting” of two-layered molecular structures, subjected to numerical density functional theory computations. It is possible to achieve high accuracy, as reported before [9], a high-throughput approximation was obtained using the CASTEP algorithm to describe the electronic structure transitions in layered molecular nanostructure materials. 

## 3. Computational Details

### 3.1. Multislice HRTEM Simulations

Computer-assisted transmission electron microscope simulations were completed using a fully dynamical calculation multi-slice method using projected potential:

(2) $$f\left(U\right)=\sum_{i=1}^{n}a_{i}e^{\left(-b_{i}U^{2}\right)}$$

where *U* represents the coordinates in reciprocal space (u, v, w) as described by Gomez-Rodríguez [15], projecting the potential over the (001) *c*-axis and adjusting *a* and *b* coefficients in a range of 98 < *a* < 128 and 96 < *b* < 128 to obtain the optimal conditions in comparison to some experimental HRTEM results, as presented in the literature [3,7]. The crystallographic structures were built using a graphical user interface as presented in the builder module of the Accelrys 6.1-Materials Studio^®^ package with crystallographic parameters as found from Joint Committee on Powder Diffraction Standards (JCPDS) data base. All honeycomb structures were created when exerting mechanical rotations at 3°, 5°, 8°, and 16°, with respect to the (001) *c*-basal axis, always keeping an inter-layer distance of 6.20 Å from molybdenum to molybdenum (Mo-Mo) in MoS_2_, 6.4 Å from tungsten to tungsten (W-W) in WS_2_ and 6.7 Å in WSe_2_ and 3.4 Å in carbon to carbon as found in carbonic rings of graphene layers.

### 3.2. Density Functional Theory Calculations

The electronic structure of each strain’s O-lattice was calculated using a seed from two large layers as built in the Materials Studio 6.1 graphical user interface; each superlattice (O-lattice) consisted of 1804 atoms approximately, as shown in Figure 1. Thus, to achieve fast conversion criteria and to reduce the computational time, the electronic calculations were performed using a representative portion called “seed”, consisting of 12 to 24 atoms, as shown in the inset of Figure 1. The “seed” was placed in a P1 (no symmetry) crystal and with varying lattice parameters from 16.0 Å ≤ *a* ≤ 18.0 Å, 17.0 Å ≤ *b* ≤ 21.0 Å, 15.0 ≤ *c* ≤ 17.0 Å and crystal lattice angles of α = β = 90° and γ = 120°. These particular distances were selected cautiously to avoid interactions with neighboring atoms in periodic cell conditions, which could yield incorrect results in both the band structure and density of states calculations. Before calculating the density of states and band structure calculations, both untwisted and twisted “seeds” were subjected to geometrical optimizations using the Broyden–Fletcher–Goldfarb–Shanno algorithm, as described by Fischer and Almlöf [16]. All DFT computations were performed using Cambridge Serial Total Energy Package (CASTEP) with a revised Perdew–Burke–Ernzerhof functional [17] general gradient approximation, a cutoff energy of 300 eV in the reciprocal space for the gamma point only with a self-consistent field (SCF) convergence threshold of 1 × 10^−6^ eV per atom and without any thermal smearing.

## 4. Conclusions

From previous investigations it is possible to achieve observations of the MoS_2_ stacked structure using experimental HRTEM; then, using dynamical multi-slice HRTEM simulations it was possible to determine a structural meaning of those honeycomb-like observations. Here we presented a more extensive study including graphene, WS_2_ and WSe_2_ layered materials, observing a new concept as presented by Bollman corresponding to the formation of a new lattice, the so-called O-lattice. To further understand the electronic structure nature of this new O-lattice, we proceeded with DFT numerical simulations where it was possible to determine some transitions and band gap reductions due to strain caused by rotating the lattices as presented here; the undergoing mechanism of those electronic states can be fully understand if a combined experiment is set by using Extended X-ray Absorption Fine Structure (EXAFS) on exfoliated 2D layered materials and with of computer assisted X-ray simulations, since we believe a crystallographic transition is clearly occurring, which can cause a space group change from hexagonal into trigonal, perturbing d-orbital clouds between layers as discussed briefly in Reference [9].

## Figures and Tables

**Table 1:** O-lattice distribution for 2D layered materials corresponding to molecular structures at various rotation angles.

Rotation Angle	Graphene	MoS_2_	WSe_2_	WS_2_
3°	12.300 Å	25.328 Å	22.974 Å	18.948 Å
5°	9.840 Å	15.830 Å	19.692 Å	15.796 Å
8°	8.639 Å	15.736 Å	14.899 Å	9.950 Å
16°	7.380 Å	12.725 Å	9.846 Å	9.474 Å
